# Ethnomedicinal plants used by the people of Manang district, central Nepal

**DOI:** 10.1186/1746-4269-2-41

**Published:** 2006-10-04

**Authors:** Shandesh Bhattarai, Ram P Chaudhary, Robin SL Taylor

**Affiliations:** 1 Central Department of Botany, Tribhuvan University, Kirtipur, Kathmandu, Nepal; 2 Department of Community Medicine, McGill University, Montreal, QC, Canada

## Abstract

**Background:**

The district of Manang (2000 – 6000 m) is located in the Central Himalayas, Nepal. The majority of local inhabitants of the area are Gurungs, of Tibetan origin. The remoteness of the region has resulted in continued use of plants as medicine in an area where the ethnobotany has sparsely been documented.

**Methods:**

Interviews were conducted with *amchi *(Tibetan medicinal practitioners), local healers (including priests locally known as *'lamas'*), plant traders, and knowledgeable villagers (including herders) regarding local plant names and their medicinal uses during several field visits (2002–2005). When convenient to the locals, a jungle or forest walk was done with the healers, allowing for both plant collection and detailed information gathering.

**Results:**

This present research documented 91 ethnomedicinal plant species, belonging to 40 families under 73 genera, and 45 new ethnomedicinal plant species are added. These 91 locally used medicinal plants are found to treat 93 ailments. This study provides information on 45 plant species previously unknown for their medicinal uses in Manang. The indication for use, mode of preparation, dose and administration of medicine are described in detail for each species.

**Conclusion:**

This wealth of ethnobotanical knowledge persists, and is being transferred to the next generation in some areas in upper Manang, in a country where this is often not the case. The senior *amchi *of the area (Karma Sonam Lama), who has been practicing Tibetan medicine for three generations, feels that it is of utmost importance to conserve the traditional healing system and to pass his knowledge on to the local community about the importance of medicinal plants. He hopes that this will lead to the conservation and sustainable management of medicinal plants in the villages. Over the duration of this research, the prices of several rare medicinal plants of Manang increased dramatically, highlighting both the scarcity and the quick disappearance of the species. This is only one example of a worrying trend of over harvesting of medicinal plants, and highlights the need for conservation and management of medicinal plants of Manang district.

## Background

The use of plants as medicine is widespread throughout the world. In many areas of rural Nepal, medicinal knowledge and practice are passed down entirely through the oral tradition and personal experience [1]. The total population of Nepal is 23.1 million [2], and about 90% of the Nepalese people reside in rural areas where access to government health care facilities is lacking. It is estimated that there is one physician for 30,000 people whereas there is one healer for fewer than 100 people in Nepal [3]. Many studies have investigated the uses of medicinal plants in Nepal [4-13], although only two studies [8,14], have been done to document the knowledge about the plants in Manang district. In the present study, a detailed ethnomedicinal survey was carried out in the Neyshang, Nar and Phoo regions of Manang district. This present research adds to that knowledge base, and this paper will be helpful to document important medicinal plants of the Nepal Himalayas in the trans-Himalayan Zone, which are often used by the Gurung communities for primary healthcare.

The district of Manang (2000 – 6000 m) is located in the Central Himalayas Nepal. The Gurungs of Tibetan origin live in the area [15].The inhabitants of Nyeshang are known by the name of Nyeshangba or, more popularly, by the name of Manangba and of Nar and Phoo by the name of Narba [16]. Manang district lies in the Annapurna Conservation Area Project (ACAP), and the permission for the field study, as well as the collection of voucher specimens was received from the headquarters of the Annapurna Conservation Area Project (ACAP) in Pokhara. Manang district is ranked in 10^th ^position among 75 districts in Nepal on overall development index [17]. The trans-Himalayan range is a unique chain of mountains with fragile ecosystems but the diversity of wild flora and fauna fulfills basic daily needs for the peoples living in the mountains and plains [18].

In Manang district herbal medicine preparations are used to treat a variety of different ailments, from cough and cold, to respiratory diseases to dyspepsia. Access to health care is often a problem in remote districts of Nepal, and Manang district is no exception. The only means of trustworthy transport in the district are chartered helicopters, or to travel by foot, which takes three days to reach to Chame, the district headquarters, from Beshisahar (Lamjung district), the road-head town. Because of the specific geographical features of Manang district, and the lack of government health facilities in the district, the people are largely dependent on the indigenous health care system. Local herbs and other plant resources found in that area are the principal source of medicine, and are prescribed by traditional healers as medicines. This is often the only source of primary health care in the district of Manang.

## Materials and methods

### Plant collection and identification

**Figure 1:**
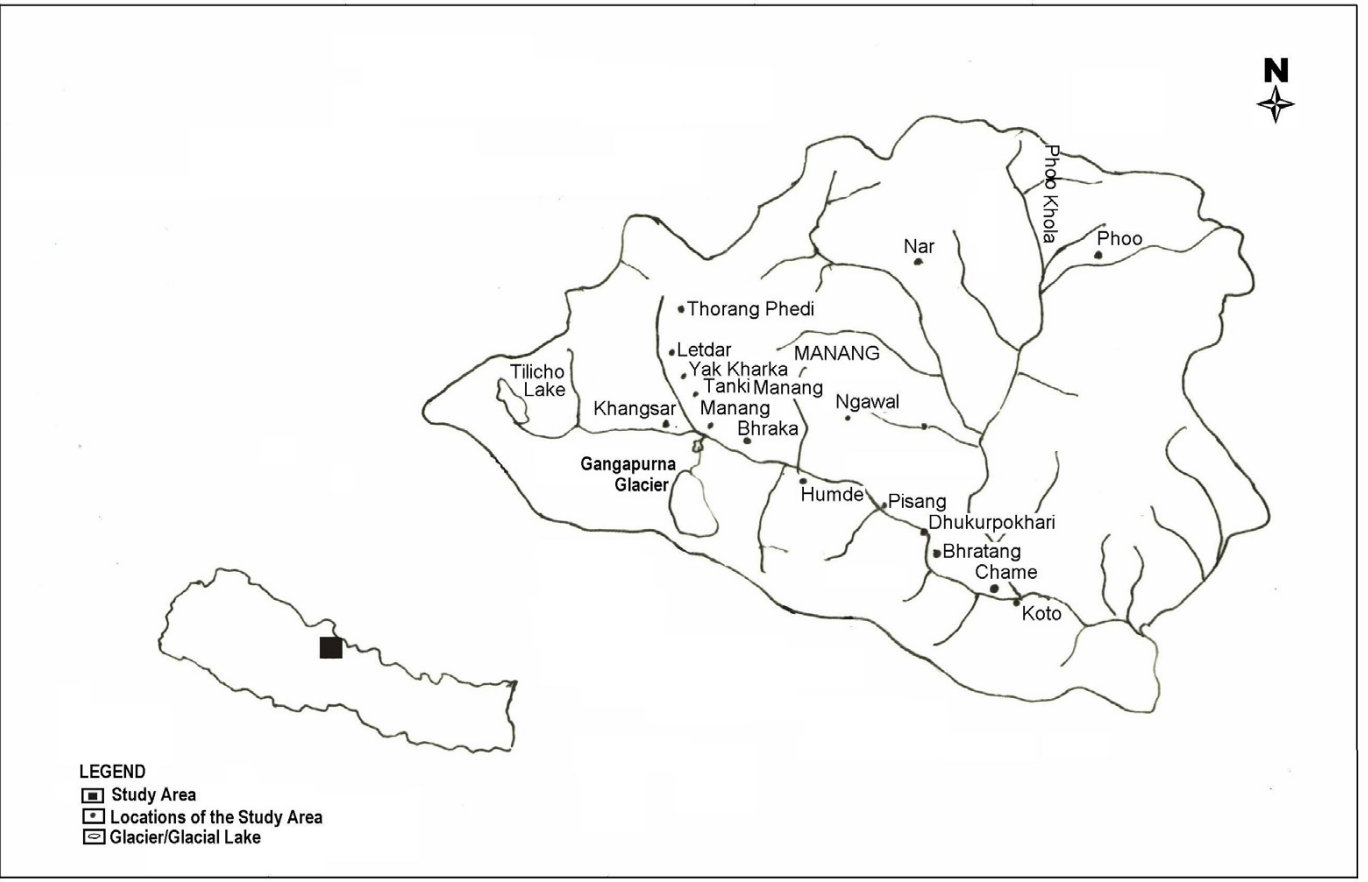
Map of the study area (Manang district), Central Nepal.

The plants were collected in and around the villages of Pisang, Hungde, Munji, Ghyaru, Ngawal, Braga, Manang, Tanki Manang, Khangshar; and places such as Tilicholake, Kecholake, Yakshed, Goatshed, Nar and Phoo in the Manang District of Central Nepal from 2002–2005 two times in each year (first in July/August, and second September/October). The study area is shown in Figure 1, Map of the study area (Manang district), Central Nepal. The district headquarters, Chame, is noted, as well as the remote villages of Nar and Phoo, which have recently opened for trekking.

Plants were selected because of their use mainly among the Gurungs of Tibetan origin. Only species that were consistently used to treat the same illness by several healers and villagers were selected. The herbarium specimens were identified by two of us (S.B. and R.P.C.) with the help of standard literature [19-23], and nomenclature of the identified species follows standard literatures [24-27]. A set of voucher herbarium specimens with voucher numbers was made for each collection and have been deposited in the Tribhuvan University Central Herbarium (TUCH) Nepal.

### Interviews with the healers

The traditional healers in upper Manang comprise *amchi *(Tibetan medicinal practitioner), local healers (that include priests locally known as *'lamas'*), plant traders, and knowledgeable villagers (including herders). Informed consent was obtained verbally from each healer or villager before they were interviewed. They were interviewed with their consent to have their names and knowledge published; and are Mukhiya Ghale, 64 years, male; Chakki Gurung, 61 years, female; Dipli Gurung, 52 years, male; Kancha Bishwakarma, 50 years, male; Kalu Gurung, 50 years, male; Karki Gurung, 67 years, male; Aoda Bishwakarma, 45 years, male; Kali Gurung, 46 years, male; Galchen Gurung, 57 years, male; Kazi Gurung, 31 years, male; Sonam Chhiring, 27 years, male; Kalu Gurung, 27 years, male; Karma Gurung, 45 years, male; Sengla Gurung, 66 years, male; Chhiring Gurung, 50 years, male; Karma Sonam Lama, 68 years, male; Lopsang Lama, 42 years, male; and Polchom Lama, 21 years, female.

Two interviewing methods were followed. The first was the 'specimen display' method. After collecting plant specimens, these species were shown to the traditional healers in order to elicit any medicinal information. The same plant specimens were shown to the different healers to confirm the accuracy of the results. When convenient to the locals, the second method of jungle or forest walk took place. A walk through the forest with the healers allowed for both plant collection and detailed information gathering.

## Results and discussion

The results of the present study are presented [see Additional file 1] with alphabetical arrangement of plant scientific name (in italics) followed by family name (in capital letters), voucher number, local names (Gurung/*amchi*/Nepali) and detailed uses (mode of preparation doses and administration of medicine). Altogether 91 plant species belonging to 40 families and 73 genera are reported from the study area. The largest number of species were noted from the families Composite (10 species), followed by Ranunculaceae (8 species), Labiatae (5 species). Three family Polygonaceae, Umbelliferae, and Rosaceae represent four plant species each. Five families, Amaryllidaceae, Liliaceae, Gentianaceae. Berberidaceae, and Boraginaceae represent three plant species each. Twelve families, including Pinaceae, Saxifragaceae, Solanaceae, Araceae, etc., were represented by two plant species each. Eighteen families including Morchellaceae, Taxaceae, Clavicipitaceae, etc., were represented by one plant species each. These 91 locally used medicinal plants are found to treat 93 ailments. Therefore, the exhaustive study of medicinal plants within the upper Manang (Neyshang and Nar-Phoo) reflects its rich diversity and history of medicinal plant use.

Although Manandhar (1987) documented 81 species of plants under 75 genera of 32 families, 45 new ethnomedicinal plants were added by the present results. Twenty-three plant species including *Cicerbita macrorhiza, Cynoglossum zeylanicum, Rhododendron lepidotum, Rosa macrophylla, Rosa sericea, Rubus foliolosus*, *Rumex nepalensis*, etc., were common ethnomedicinal plants in the above and the present results; however, additional uses were also noted in the present results. Pohle (1990) documented 239 species of plants, of which 66 species of plants were used ethnomedicinally. Compared with Pohle (1990), the present results added 60 new species of plants which were used ethnomedicinally in the same area. Thirty-one plant species were found to have the same ethnomedicinal uses as documented by Pohle, but the present investigation finds more uses of those same plants. When comparing both Manandhar (1987) and Pohle (1990) to the present results, 45 plant species are newly documented for their ethnobotanical usage. Those newly added plant species are noted with asterisks (*) [see Additional file 1]. It is hoped that these newly documented plants may be valuable for future research activities. This initial documentation was the first step of bioprospecting of medicinal plants of Manang. Perhaps further steps in bioprospecting with regards to these medicinal plants will be beneficial for the discovery of new drugs/medicines.

When the present results are compared to the Tibetan literature [28] 72 ethnomedicinal plant species were newly recorded and 19 plant species including *Mirabilis himalaica*, *Fragaria nubicola*,*Arisaema flavum*, *Arisaema jacquemontii *etc., were found to have the same ethnomedicinal results. Comparison to Ayurvedic literature [29] showed that 83 species documented here are new. The eight species that had known Ayurvedic uses are commonly used in Manang: *Berberis aristata*, *Cannabis sativa*, *Carum carvi*, *Ephedra gerardiana*, *Nardostachys grandiflora, Neopicrorhiza scrophulariiflora, Rhododendron anthopogon *and *Valeriana jatamansii.*

This study found that many different parts of the medicinal plant species are used as medicine (namely as flowers, leaves, root, stem, whole plant, fruits, seeds, bark, latex, and cones) but the most commonly used plant part was flowers (used in 31 species), followed by leaves (30 species), root (26 species), stem (17 species), whole plant (14 species), fruits (11 species), seeds (7 species), bark (7 species), latex (3 species), and cones (1 species). It would follow that these plant parts have been selected because vulnerable flowers, leaves, and roots may contain more active principles in comparison to fruits, seeds, bark, and latex. Leaves, roots, stems and flowers are physically more vulnerable than bark or cone, and therefore it is not surprising that they contain more chemical defence compounds in the form of biologically active secondary metabolites.

Medicines are prescribed in different forms including powder, paste, decoction (liquid obtained from boiling or the medicinal plants in the solvent), and infusion (plant powder/paste mixed with the solvent). In this study, powders and decoctions were found to be used more often in comparison to pastes and infusions. Medicines are prescribed in both ways, as a single drug and in mixed ingredient form. In mixtures, several to many valuable medicinal plants are mixed with the other (often local) medicinal plants in standard amounts. The mixture is not changed depending on the person but the dose may be changed with age. Only herbal treatments were considered for this paper, leaving animal and mineral treatments for possible future research. Out of the 91 plant species, 25 species are used to cure only one disease and the remaining 66 plant species are used to cure more than one disease. A detailed list of these ailments can be seen in Table 1.

**Table 1:** Lists of ailments treated by local traditional healers in Manang district, Central Nepal, grouped by body system.

**Body System (Categories)**	**Ailments treated by the local traditional healers**
Skin	warts, wound problems (infections), wounds on skin, abrasions, skin swelling, boils, scabies, cuts and wounds, burns, ringworm, and blisters
Cardiovascular	heart disease, heart pain
Respiratory	shortness of breath, sore throat, chest pain, asthma, bronchitis, reduce sound production (wheezes or stridor) during breathing, tuberculosis
Neurological	numbness of limbs, paralysis, pulse pain, and 'vein pain'
Reproductive	infertility, wounds in vagina 'Bhringhee', quicken labour and delivery, stop bleeding during child birth and to increase sexual power
Gastointestinal	diarrhoea, dysentery, vomiting, stomachache, gastritis, worms (white intestinal worms), anthelmintic, bile disorders, constipation and stomach ache
Orthopaedic	Heal broken bones, bone fracture, back pain, fracture of hand and leg, bone pain, bone diseases, joint pain
Blood	Increases the blood, purification of blood, menstrual disorders (heavy flow), menstrual problems, high blood pressure and blood circulation
Renal/Urological	Kidney diseases, urinary tract infection, diuretic, dysuria.
Muscular	Increase body size, for body massage, 'waist pain', inflammation of body, rheumatism, neck pain, and limbs pain
ENT	conjunctivitis, pain in nose (internal or external), eye pain, stop bleeding from nose, eye diseases, blindness, defects in visions, ear pain, sinusitis, gingivitis, relief tooth pain, cough and cold, and tonsillitis,
Paediatric	massage the head of children
Other/ Whole body/ Systemic	fever (any kind of fever i.e., typhoid fever, malaria), jaundice, headache, high altitude sickness, vertigo/ dizziness, stop sweating, diabetes, cancer, snake bite and scorpion sting, vitamin, nutritious, tonic (to treat weakness), infectious diseases, rib pain (*Kokho dukhnu*), tuberculosis, edema (swelling of the body), body pain, and pain from swelling

Plant parts were generally prepared as medicine using hot and cold water as the 'solvent', but occasionally remedies were prepared with milk, honey, oil and ghee. It is interesting and important to mention that medicines are sometimes prepared with ghee (butter from a female Himalayan cow) when a patient has fever. For example *Aconitum naviculare *is used for fever and jaundice. Half spoonful of powder (made from dried whole plant) is mixed with 2 spoonfuls of Chauri ghee (butter from a female yak) and taken two times a day for fever and jaundice until recovery. This is a unique method of treatment of the fever patient. This local tradition was explained as to have started to 'sweeten' the taste of bitter *Aconitum naviculare*. This is interesting as doctors in Nepal often prohibit the use of oil for cooking when the patient has fever.

The more common use of water in the preparation of medicine could be due to reduced availability of other infusion materials such as milk, honey, oils and ghee in the rural villages. Milk and ghee are expensive to buy if a family does not own the animals. This does not explain the use of milk as a solvent. Water and milk have different properties and milk, containing fats, will dissolve chemicals that water will not. Perhaps some plants are mixed with milk to free chemicals that are not water soluble.

Medicinal plant use in Manang is not restricted to local plants. The *amchis *of Manang import several valuable medicinal plants from the lower regions of the district including the Terai (southern plains) region for the preparation of medicine. These include such as *Rouvolfia serpentine*, *Phyllanthus emblica*, *Terminalia bellirica*, and *Terminalia chebula*. Many valuable medicinal plants of Terai are known to the *amchis *and many are also mentioned in the Tibetan medical literature. An *amchi *will always collect local medicinal plants himself/herself. They stress that this is important because they have the experience to identify the plants. They worry that a misnamed or falsely collected sample may be dangerous for the patient.

*Amchis *may also create confidential mixtures of medicines for a patient. This confidentiality was maintained and no data regarding those mixtures were recorded. When permission was granted, the plants species has been indicated (for example [see Additional file 1]) with the confidential nature of the mixture noted. Many plant species are used by *amchis *to treat a broad range of ailments, while some plant species are found to treat only one aliment. For example, *Allium oreoprasum*, *Aster diplostephioides*, and *Neopicrorhiza scrophulariiflora *treat broad range of ailments, while *Maharanga bicolour*,*Maharanga emodi*, etc., are used to treat only one ailment (ear pain). In total, 91 medicinal plants were found to be used to treat 93 ailments. The most commonly treated ailments include gastritis, cough and cold, edema, stomachache, diarrhoea, dysentery, jaundice, rheumatism (disease characterized by inflammation of joints, muscles or connective tissue), and numbness of limbs.

**Figure 2:**
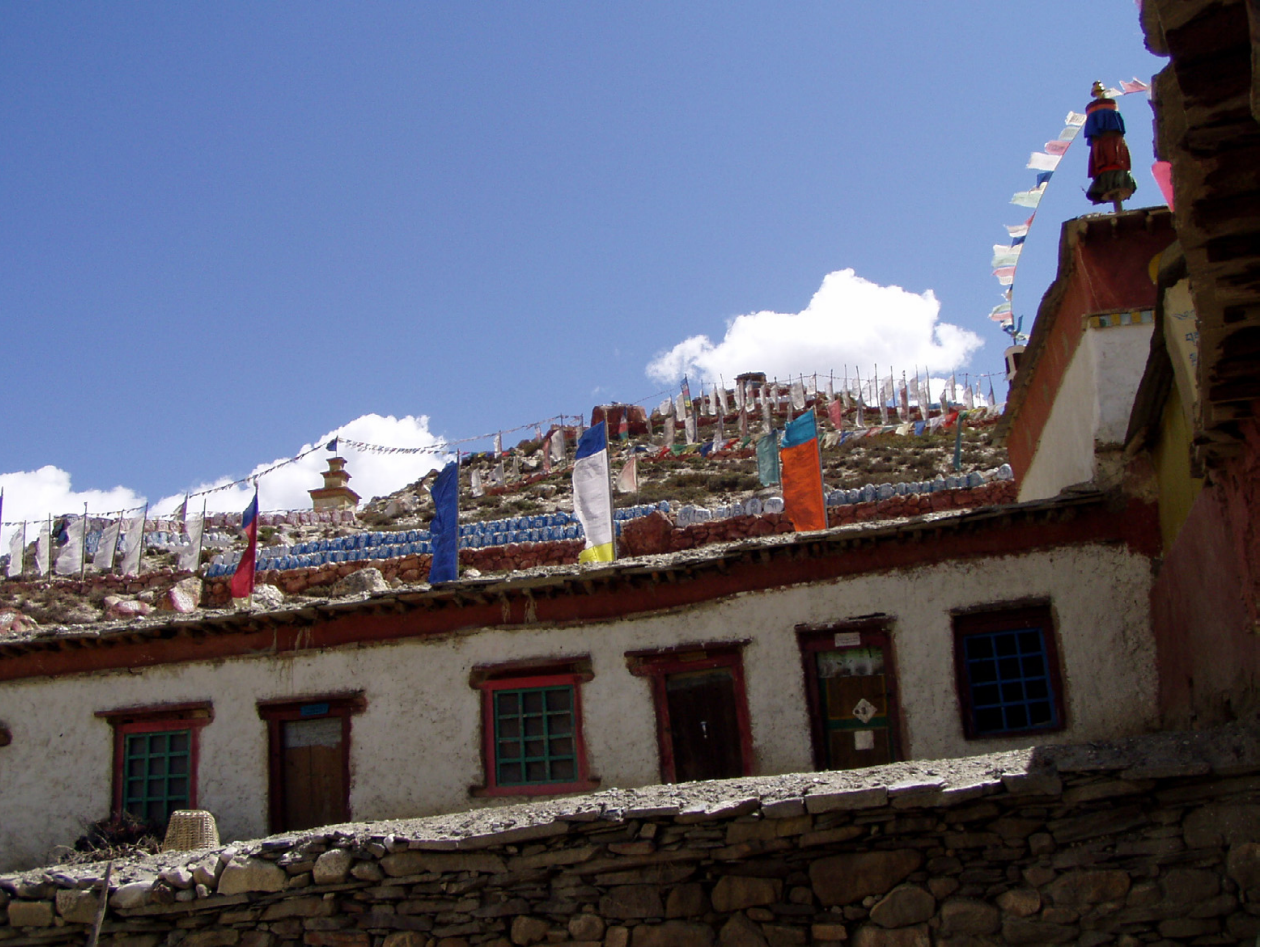
Monastery of Phoo

The health care system in Manang district is basic. Few allopathic medicines are available, with the exception of paracetamol (acetaminophen) and bruffin (ibuprofen). For this reason, and because of traditional and cultural customs and beliefs, many people rely on local healers or *amchis *for health care. The *amchis *have great knowledge about the use of medicinal plants, and the villagers rely heavily on them, and so are not without health care. Interestingly, not only do the people of Nar and Phoo depend on *amchis *for health care, but patients from surrounding villages come to the village of Phoo, the site of a monastery with much respected *amchis*, for treatment. Figure 2 shows the Monastery of Phoo. For example, one patient (who agreed to the use of her story as long as her name was not used) from Tilche (of lower Manang village), travelled to the monastery for the treatment of dysfunctional vaginal bleeding. She had sought treatment in several places in Nepal and in India but her condition had not improved. The *amchi *gave a confidential mixture, prepared especially for this patient, continuously for a week. After the treatment the patient recovered and returned to her home. This account echoes the trust and respect that the peoples of Manang district have for the traditional healers and *amchis *for primary health care.

The remote village of Phoo, where this *amchi *practices, contains only 28 houses; and from these houses, 23 people are now working outside the country after having been trained by the most senior *amchi *(Karma Sonam Lama). It is very easy to leave the country after completing this training, as healers trained by this senior *amchi *are sought after. Both women and men come to the monastery to seek training.

The senior *amchi *is also familiar with the very intricate rules for collecting plants for use as medicines, such as time of collection, parts to be collected, and care in the conservation and management aspects of medicinal plants. He feels that it is of utmost important to conserve the traditional healing system and aware the local community about the importance of medicinal plants which will lead them to the conservation and management of medicinal plants in the villages. If this healing system cannot be conserved and utilized, he feels that in time it will have a negative effect on the whole community. He feels fortunate that his son, Lopsang Lama (age 42 years) and his daughter, Polchom Lama (21 years) are learning the traditional healing system from their father, and are acquiring a deep understanding of the use of medicinal plants. Many plant species of Manang district are used for medicine, several are very popular and known throughout Nepal.

Two species of *Hippophae *are included in this group of important medicinal plants. *Hippophae salicifolia *fruit is used for cough and cold, chest pain, stomachache, diarrhoea, dysentery, worms, rheumatism and gastritis and *Hippophae tibetana *fruit is used as a diuretic, tonic, cough and cold, to treat periods of weakness, and worms. These two species are used to treat broad range of ailments. It is very important to take immediate steps towards conservation and management aspects of these two important medicinal plants of Manang. Cultivation of these two species in the barren lands helps in conservation and management, while at the same time can improve the economic status of poorer people. Several development projects use fruits to make a now popular juice in the districts of Manang and Mustang, commonly called 'Seabuckthorn juice'.

Several medicinal plants are collected not just to be used locally, but also to sell to supplement earnings of the local people. Healers, *amchis*, local people and people of Gorkha district who come to Manang to earn their livelihood collect highly prized medicinal plants including *Allium oreoprasum*;*Cordyceps sinensis*; *Dactylorhiza hatagirea*; and *Neopicrorhiza scrophulariiflora*.

Collection of the protected medicinal plant *Cordyceps sinensis *(Yartsagumba) is increasing these days in Manang. It has been banned for export to foreign countries except after processing within the country and with the permission of the Department of Forests. Collectors of *Cordyceps sinensis *stay for a month or two (May-June) in the Yakshed region, over 4500 m above sea level, in the areas of Kecho Lake (Icelake area), Khangshar, Braka, Hungde, Nar and Phoo. *Cordyceps sinensis *is sold for 34–45 Rupees/piece (equivalent to US $0.50) to local healers, and local medicinal plant sellers. The local healers and *amchis *use it to make medicine, while plant sellers take it to Kathmandu and/or Pokhara and sell it for double or triple the price it sells for in Manang. Over the duration of this field research, (from 2002–2005) the going rate of one piece of *Cordyceps sinensis *increased from NRs 25 to 85 (equivalent to US$1.25). This sudden increase in the price of this highly used medicinal plant highlights both their scarcity and the quick disappearance of the species. This is only one example of a worrying trend of over harvest of medicinal plants, and highlights the need for conservation and management of medicinal plants of Manang district.

## Conclusion

The present survey concludes that the local population of Manang district has a fairly extensive and detailed knowledge regarding wild plants and their utility. The inhabitants of Nyeshang, Nar and Phoo in Manang district are ethnobotanically very rich. They have a wide knowledge on the use of plants for various purposes, including medicinal, food, fuel-wood, fodder, timber, household article, incense, etc., With regards to the uses of plants as medicines, this research confirms the vast knowledge of the traditional healers such as *amchis*, local healers and village elders on the subject of plants used for medicinal purposes.

Several factors may contribute to the persistence of this knowledge. The lack of modern and government facilities and remote geographical features of Manang district, as well as a strong belief in folk medicines continue the preference for traditional healers for their health care. This tradition is strong in the remote villages of Nar and Phoo, where the *amchi *is the only source of primary health care. Generally, most village elders can treat minor diseases themselves, using local medicinal plants. If they cannot treat the illness by themselves, they will seek help from the senior *amchi *(Karma Sonam Lama) of Phoo. There are other more junior healers and traditional medicinal plant sellers in the village of Phoo, but none more expert than the senior *amchi*.

The senior *amchi *of Phoo is grateful that his children are interested in learning how to use medicinal plants. It is common in the area to have a lack of flow of indigenous knowledge from elder to younger generation, since the young generation is reluctant to learn about traditional medicinal practices. The younger generation often leave their villages because of the profound economic changes that have come about during the last ten years. Many young families have left Manang and established themselves in the cities of Kathmandu or Pokhara.

In the quest to increase earnings, important medicinal plants are now being harvested for profit, which may put some species at risk. For example large amounts of *Cordyceps sinensis *are collected each year in the district. The continuation of collection of *Cordyceps sinensis *in the same area each year will decrease the population of the species and the locals worry that plants are rarer now and the species disappearing from the district. Indigenous practices and knowledge regarding the sustainable harvest and utilization of plant resources as medicine should be documented and preserved before they disappear.

## Competing interests

The author(s) declare that they have no competing interests.

## Authors' contributions

Author SB performed the interviews with the healers, identified the herbarium specimens with RPC and drafted and finalized the manuscript with RPC and RSLT. Author RPC joined SB to perform interviews, identified herbarium specimens with SB, supervised the research works and finalized the manuscript with SB and RSLT. Author RSLT supervised the research works and drafted and finalized the manuscript with SB and RPC.

## Supplementary Material

Additional file 1 Ethnomedicinal plants used by the people of Manang District, Central Nepal. An alphabetical listing of local medicinal plants by species, with family, voucher number and local vernacular name mentioned, as well as a detailed description of preparation and use. *indicates species not previously known for its medicinal use in Manang Click here for file
