# Supplementary material for: Ethnomedicinal plants used by the people of Manang district, central Nepal
Source: J Ethnobiol Ethnomed. 2006 Oct 4;2:41. doi: 10.1186/1746-4269-2-41 (PMC1618386; doi:10.1186/1746-4269-2-41)
Supplement: Additional file 1 — Ethnomedicinal plants used by the people of Manang District, Central Nepal. An alphabetical listing of local medicinal plants by species, with family, voucher number and local vernacular name mentioned, as well as a detailed description of preparation and use. *indicates species not previously known for its medicinal use in Manang [file 1746-4269-2-41-S1.doc]

**Appendix: Ethnomedicinal plants used by the people of Manang District, Central Nepal.**

An alphabetical listing of local medicinal plants by species, with family, voucher number and local vernacular name mentioned, as well as a detailed description of preparation and use.

*indicates species not previously known for its medicinal use in Manang

1) **Abies spectabilis* (D. Don) Mirb. (PINACEAE), Voucher 342. ‘Kye’ (Gurung), ‘Thangwha’ (Amchi).

a) To heal broken bones, fresh leaves and cone are pulverised on a stone slab and the paste is applied to the site of the fracture two times a day until recovery. This is not used on compound fractures that have broken the skin.

b) Also for bone fractures approximately 20g cones and leaves are mixed with two cup of water and boiled it for sometime. Half a cup of decoction is again mixed with a cup of hot water and drunk 1-2 times a day until recovered. If the patient is suffering from fever (fever due to bone fracture) then the decoction is mixed with cold water instead of hot.

2) *Aconitum naviculare* (Bruhl) Stapf (RANUNCULACEAE), Voucher 295. ‘Bhalaponkar’ (Gurung).

a) About 15g of whole plant is pounded on stone slab and boiled on two cups of water. About 5-10 spoonful of decoction is mixed with a cup of water and the decoction is drunk two times a day after meal for fever (any kind of fever) and jaundice. It is necessary to take a ‘vitamin’ tonic of ‘Yartsagumba’ (*Cordyceps sinensis)* and ‘Lovha’ (*Dactylorhiza hatagirea*) after using the medicine. It is said that the medicine works as an antibiotic. Regular use of this medicine causes weakness, so in order to counteract that, as well as to make sweeten the mouth, equal amounts of ‘Yartsagumba’, ‘Lovha’, and cow’s milk sweetened with honey are taken as a source of ‘vitamins’. This mixture is known as the best type of ‘vitamin’ of that area.

b) Half spoonful of powder (made from dried whole plant) is mixed with a cup of boiled water and drunk 2-3 times a day for fever, and jaundice until recovery.

c) Half spoonful of powder (made from dried whole plant) is mixed with 2 spoonfuls of Chauri ghee (butter from the female cow) and taken two times a day for fever, and jaundice until recovery.

3) *Aconitum orochryseum* Stapf (RANUNCULACEAE), Voucher 2032. ‘Nirmasi’ (Gurung).

a) Half a spoonful of ground root powder is taken with a cup of hot water 2-3 times a day until recovery for fever, diarrhoea, dysentery, cough and cold, tonsillitis, headache and high altitude sickness problems. The symptoms of high altitude sickness include vomiting, vertigo/dizziness (‘*ringhata lagnu*’), headache, eyes becoming red and blue due to extreme cold, and shortness of breath. It is necessary to take a ‘vitamin’ tonic of ‘Yartsagumba’ (*Cordyceps sinensis)* and ‘Lovha’ (*Dactylorhiza hatagirea*) after using the medicine. It is said that the medicine works as an antibiotic. Regular use of this medicine causes weakness, so in order to counteract that, as well as to make sweeten the mouth, equal amounts of ‘Yartsagumba’, ‘Lovha’, and cow’s milk sweetened with honey are taken as a source of ‘vitamins’. This mixture is known as the best type of ‘vitamin’ of that area.

4) *Allium carolinianum* DC. (AMARYLLIDACEAE), Voucher 2179. ‘Rotangtea’ (Gurung).

a) Fresh parts of whole plant are pounded on a stone slab and 1-2 spoonfuls are used as a spice substitute in curry dishes (vegetable or meat curry). The dish is eaten two times a day for stomachache, headache, diarrhoea, and dysentery until recovery.

b) One-forth spoonful of powder (made from dried whole plant) is drunk with a cup of boiled water two times a day for the same diseases mentioned above. The whole plant is also given as a meat substitute to a recently post-partum mother (with a baby of 3-12 days) because it is highly nutritious, fulfils the requirement of meat and ‘increases the blood’.

5) **Allium fasciculatum* Rendle (AMARYLLIDACEAE), Voucher 258. ‘Nosyante’ (Gurung).

a) About 10g of whole plant is pounded and boiled by mixing with two cups of water. Half cup of decoction is drunk two times a day to reduce gastritis and to increase and purify the blood.

6) **Allium oreoprasum* Schrenk (AMARYLLIDACEAE), Voucher 2104. ‘Lungho’ (Gurung).

a) One spoonful of dried whole plant by cutting it in small pieces is put on one spoonful of mustard oil and added to pulse/lentil and/or vegetable dishes as a spice to treat cough and cold, headache, tonsillitis, stomachache, white intestinal worms, and high altitude sickness.

b) Half a spoonful of the same powder (made from dried whole plant) is taken with a cup of hot water two times a day for the same diseases mentioned above. An over dose can give vertigo/dizziness (‘*ringhata lagnu*’).

7) *Anaphalis triplinervis* (Sims) C.B. Clarke (COMPOSITAE), Voucher 139. ‘Fojormendho’ (Gurung), ‘Tayung’ (Amchi).

a) The whole dried plant is ground to a powder. Half spoonful of powder is put on red fire coal and the scent/smoke is sniffed by the patient two times a day after meals for cough and cold, and tonsillitis.

b) About 10g of whole plant is boiled in one cup of water and 10 spoonful of decoction is drunk two times a day after meal for cough and cold, tonsillitis and fever.

c) One-forth spoonful powder (made from dried whole plant) is taken 2-3 times a day after meal for fever, menstrual disorders (heavy flow), and edema (swelling of the body), until recovery.

d) The paste of leaves and flowers is applied daily on abrasions that have become inflamed, infected and/or swollen. The abrasion is then ‘dried’ by placing the affected body part close to a fire once a day to ‘remove water’, until recovery.

8) *Androsace strigillosa* Franch. (PRIMULACEAE), Voucher 169. ‘Gadhikanakyo’ (Gurung).

a) Half spoonful of powder (made from dried whole plant) is taken with a cup of boiled water two times a day after meal for edema (swelling of the body), and/or fever, until recovery.

b) The paste of the whole plant is applied on skin and then dried with a red fire coal to remove water from the skin that occurs with ‘skin swelling’. This ‘skin swelling’ refers to the accumulation of water inside the skin in the cold seasons in the hands and legs where there has been an abrasion.

9) *Anemone rivularis* Buch.-Ham. ex DC. (RANUNCULACEAE), Voucher 492. ‘Angsoup’ (Gurung).

a) Half spoonful of ground powder (made from dried whole plant) is taken with a cup of boiled water for cough and cold, white intestinal worms, stomachache, and edema (swelling of the body), until recovery.

10) *Anisodus luridus* Link & Otto (SOLANACEAE), Voucher 159. ‘Langtang’ (Gurung).

a) Dried flowers are cut into small piece and mixed with tobacco taken from inside a cigarette. The mixture is rubbed between the palm and thumb and rolled into a cigarette. The cigarette is smoked two times a day for gingivitis and to relieve tooth pain.

11) **Arisaema flavum* (Forssk.) Schott (ARACEAE), Voucher 618. ‘Timtry’ (Gurung), ‘Tangdhung’ (Amchi).

a) The root tuber is put on fire coal under ash and one-forth part is taken one time a day for skin diseases, wart, and edema (swelling of the body), and wounds on skin, and in the vagina (‘*Bhringhee*’) until recovery.

b) The root tuber after put on fire coal under ash is used as paste on the infected part for the same diseases mentioned above until recovery.

12) **Arisaema jacquemontii* Blume (ARACEAE), Voucher 206. ‘Thomo’ (Gurung), ‘Dhaba’ (Amchi).

a) One-forth of a root tuber is eaten to treat pain of the nose (internal or external), eye pain, and warts. The tuber is baked in the hot coals from a fire and eaten once a day until recovery.

b) The paste of a roasted (‘*polnu*’ in Nepali) root tuber is applied on the infected part of the body one time a day until recovery. It can be applied inside the infected part as well outside the infected part as long as it has been properly prepared (‘*Polnu*’) on red fire coal other wise it is poisonous.

13) **Artemisia gmelinii* Weber ex Stechm. (COMPOSITAE), Voucher 138. ‘Bajha’ (Gurung).

a) The plant parts (leaves stem and flowers) is collected and ground to make powder. Half spoonful of powder is mixed with a cup of boiled water 2–3 times a day after meal to treat fever.

b)About 10g of plant parts (leaves, stem and flowers) are boiled in two cups of water and five spoonfuls of this decoction is drunk with a cup of milk 2-3 times a day after meals for fever, cough and cold, and sore throat.

14) **Asparagus filicinus* Buch.-Ham. ex D. Don (LILIACEAE), Voucher 2125. ‘Nirshing’ (Gurung).

a) One spoonful of root powder is taken once a day after a meal with a cup of hot water for menstrual disorders and menstrual problems; as a tonic; to increase body size, to stop bleeding from nose, heart diseases, and stomach disorders until recovery.

b) One spoonful of pounded root is mixed with two glass of milk and boiled it until it becomes one cup. The one cup of decoction is drunk once a day after a meal for the same conditions mentioned above.

c) The paste of the root is applied two times a day for skin diseases until recovery.

15) **Aster diplostephioides* (DC.) C.B. Clarke (COMPOSITAE), Voucher 203. ‘Mara’ (Gurung), ‘Motolugmick’ (Amchi).

a) About 10g of flower is boiled in 2 cups of water and one cup of the decoction is drunk two times a day after meal for cough and cold, headache and sore throat.

b) The flower is ground to a powder, and half a spoonful of powder is mixed with a cup of boiled water two times a day for cough and cold, tonsillitis, and/or headache.

c) Half spoonful of flower powder is taken with a cup of hot water two times a day after meal for snake bite and scorpion sting, wound problems, chest, backbone pain, pulse pain (‘*nadhidhukhunue*’) and numbness of limbs until recovery.

16) **Aster stracheyi* Hook. f. (COMPOSITAE), Voucher 415. ‘Mara’ (Gurung).

a) About 10g of pounded flower is mixed with a cup of boiled water. Half cup of decoction is drunk two times a day for cough and cold until recovery.

b) Half a spoonful powder (made from dried whole plant) is mixed with a cup of milk, and drunk two times a day after meal for cough and cold, headache and fever until recovery.

17) **Astilbe rivularis* Buch.-Ham. ex D. Don (SAXIFRAGACEAE), Voucher 2074. ‘Bhadhangoo’ (Nepali).

a) One spoonful of ground root powder is taken with a cup of hot water or milk once a day for fever, vertigo/dizziness (‘*ringhata lagnu*’), headache, and infertility, until the patient has recovered.

18) **Berberis angulosa* Wall. ex Hook. f. & Thomson (BERBERIDACEAE), Voucher 444. ‘Kyunudzu’ (Gurung).

a) About 15g of root is boiled on two cups of water and five spoonful of decoction is drunk 2-3 times a day after meal for cough and cold, fever, and/or dysentery.

b) Half spoonful of root powder is mixed with a cup of milk and drunk 2-3 times a day after meal for fever.

19) **Berberis aristata* DC. (BERBERIDACEAE), Voucher 498. ‘Karya’ (Gurung).

a) The decoction of the paste of flowers leaves and bark is applied on the body for edema (swelling of the body), 2-3 times a day until recovery.

b) One - two drops of filtered decoction of flower is put on the eye for eyes diseases (eye infection i.e., eye pain, and conjunctivitis) until recovery.

20) *Berberis ceratophylla* G. Don (BERBERIDACEAE), Voucher 353. ‘Kyerpa’ (Gurung).

a) About 10g of pounded bark is mixed with two cups of boiled water and 10 spoonful of decoction is mixed with a cup of milk and taken 2-3 times a day for fever.

b) Bark is ground to make a powder. Half spoonful of powder is mixed with two spoonful of Chauri-ghee two times a day for fever.

21) *Bergenia ciliata* (Haw.) Sternb. (SAXIFRAGACEAE), Voucher 2070. ‘Pakhanved’ (Gurung, Nepali), ‘Khadur’ (Amchi).

a) Half spoonful of ground root powder is taken with a cup of hot water two times a day after meals for diarrhoea, dysentery, stomachache, and blindness, until recovery.

22) *Betula utilis* D. Don (BETULACEAE), Voucher 201. ‘Buspath’ (Gurung).

a) Bark and leaves are ground to make powder and mixed with other different medicinal plants of the Himalayas (confidential mixture because they mix many kinds of plants). Half spoonful of powder is mixed with two spoonful of cow ghee, and taken two times a day for fever until recovery.

23) **Bistorta affinis* (D. Don) Greene (POLYGONACEAE), Voucher 203. ‘Khaldi’ (Gurung).

a) The root is collected and pounded on a stone slab. About 10 g of this pulverized root is boiled with a cup of water and half cup of the decoction is drunk 2-3 times a day for cough and cold, tonsillitis, and fever until recovery.

b) The root is ground to powder. Half a spoonful of the powder is mixed with a cup of boiled water and drunk 2-3 times a day for the same diseases mentioned above.

24) **Bistorta macrophylla* (D. Don) Sojak (POLYGONACEAE), Voucher 132. ‘Khaldhi’ (Gurung).

a) About 10g of root is pounded on a stone slab and mixed with a cup of boiled water. Five spoonfuls of this decoction is mixed with a cup of milk and drunk two times a day for typhoid fever until recovery.

b) The root is ground to make powder. Half spoonful of powder is taken with a cup of boiled water 2-3 times a day after meal for typhoid fever until recovery.

25) *Bupleurum longicaule* Wall. ex DC. (UMBELLIFERAE), Voucher 221. ‘Mirmire’ (Gurung).

a) About 10g of flowers and seeds were boiled in two cups of water and one cup of the decoction is drunk two times a day for cough and cold, and tonsillitis until recovery.

26) **Cannabis sativa* L. (CANNABACEAE), Voucher 615. ‘Kantsya’ (Gurung).

a) A pinch of flower and seed powder is taken with a cup of boiled water for stomachache, constipation, or urinary tract infection (urine becomes yellow-red in color and with a bad smell- said to be caused by ‘increased blood’ in the body). An overdose of this tea will cause vertigo/dizziness (‘*ringhata lagnu*’), and headache.

27) **Caragana brevispina* Royle (LEGUMINOSAE), Voucher 197. ‘Momosing’ (Gurung).

a) Half spoonful of stem powdered is taken two times a day after meal for cough and cold, skin diseases, heart pain, or defects in vision, until recovery.

28) *Carum carvi* L. (UMBELLIFERAE), Voucher 181. ‘Chir’ (Gurung).

a) About 20g of seeds are pounded on stone slab and boiled it on one cup of water and half cup of decoction is drunk two times a day for cough and cold until recovery.

b) About 10-30 seeds are boiled in mustard oil and massaged on the head for headache and when the body feels cold. It is mostly used for infants less than a year old.

29) *Cicerbita macrorhiza* (Royle) Beauv. (COMPOSITAE), Voucher 272. ‘Mendho’ (Gurung).

a) Half kg of root is cooked with 1 kg of wheat flour for half an hour with 10 Litres of water and given to the animals for fever one time a day until recovery.

30) *Clematis barbellata* Edgew. (RANUNCULACEAE), Voucher 112. ‘Kramay’ (Gurung).

a) The leaf and flower paste is applied around boils, scabies, cuts and wounds, fracture of hand, leg and massages to relieve back pain and ‘waist’ pain.

b) Half spoonful of leaf and flower powder is taken two times a day after meal for cough and cold and body pain, until recovery.

31) *Clematis tibetana* Kuntze (RANUNCULACEAE), Voucher 52. ‘Damongnakyo’ (Gurung).

a) Half spoonful of ground powder of plant leaves, stems and flowers is taken with a cup of hot water two times a day for cough and cold, and tonsillitis until recovery.

b) The paste of the plant parts (leaves, stems and flowers) is applied on wounds two times a day until recovery.

32) **Clinopodium umbrosum* (M. Bieb.) C. Koch (LABIATAE), Voucher 155. ‘Sarshang’ (Gurung).

a) The plant parts (leaves, stems and roots) are ground to make powder. Half spoonful of powder is put on red fire coal and the scent is smelled by the person affected from paralysis, high blood pressure, pain and inflammation of body, and difficulty moving hands and legs (but not paralysis), two times a day until recovery.

33) *Cordyceps sinensis* (Berk.) Sacc (CLAVICIPITACEAE), Voucher 518. ‘Yartsagumba’ (Gurung).

a) Half spoonful of ‘yartsagumba’ powder (from dried whole plant) is mixed with a cup of milk or honey and used during the periods of weakness when taking the ‘antibiotics’ (*Aconitum naviculare,/ Neopicrorhiza scrophulariiflora*) of that area as a tonic once a day until recovery.

b) Half spoonful of ‘yartsagumba’ powder (made from dried whole plant) + half spoonful of *Dactylorhiza hatagirea* powder (made from root) + half cup of honey or milk is mixed and used as tonic during enervate periods.

c) One piece of ‘yartsagumba’ is put in one cup of local home made alcohol and drunk continuously in the morning and evening by male as a tonic (they believe that it serves as tonic).

34) **Cynanchum canescens* (Willd.) K. Schum. (ASCLEPIADACEAE), Voucher 2034. ‘Dhugmoyung’ (Gurung).

a)Half spoonful of ground powder (made from leaves and flowers) is taken with a cup of boiled water 2-3 times a day for cough and cold, diarrhoea, dysentery, kidney diseases, fever, and stomachache, until recovery.

b) Two to four drops of the fresh juice of the flowers and seeds are put on ear for ear pain 2-3 times a day until recovery.

35) *Cynoglossum zeylanicum* (Vahl) Thunb. ex Lehm. (BORAGINACEAE), Voucher 145. ‘Thina’ (Gurung).

a) The flowers are pounded on a stone slab and the paste is applied around boils. It helps to draw out pus and quickens the healing process.

36) *Dactylorhiza hatagirea* (D. Don) Soo (ORCHIDACEAE), Voucher 447. ‘Panchaaule’ (Gurung), ‘Lovha’ (Amchi).

a) The paste of the root is applied around boils, cuts and wounds, burns, scabies, ringworm, snake and scorpion stings once a day until recovery.

b) Half spoonful root powder of (‘Lovha’) *Dactylorhiza hatagirea* is mixed with half spoonful powder of ‘yartsagumba powder’ (*Cordyceps sinensis*) made from whole plant and then mixed both powder again with a cup of honey or milk. This can be used as a general health tonic once a day until recovery.

37) **Delphinium brunonianum* Royle (RANUNCULACEAE), Voucher 261. ‘Ponmar’ (Gurung).

a) About 10g of whole plant is boiled in 2 cups of water and half cup of decoction is drunk two times a day after meal for jaundice and fever, until recovery.

38) **Delphinium stapeliosum* Bruhl ex Huth (RANUNCULACEAE), Voucher 255. ‘Ponmar’ (Gurung).

a) About 10g of pounded whole plant is mixed with two cups of water, then five spoonful decoction is mixed with a cup of milk two times a day after meal for fever (typhoid, malaria), and jaundice. It acts as an antibiotic, and is said to result in a lack of energy in the patient. So it is necessary to take vitamin or tonic (Yartsagumba + Lovha).

b) Half spoonful powder (made from whole plant) is mixed with three spoonful of Chauri ghee and taken two times a day after meal for fever.

39) *Dicranostigma lactucoides* Hook. f. & Thomson (PAPAVERACEAE), Voucher 105. ‘Rhafendhi’ (Gurung).

a) About 500g, root is collected and pounded on stone slab. It is cooked by mixing it with wheat flour and rice for 15 minutes, and then given to the animals which are going to have a baby to quicken labour and delivery.

40) *Elsholtzia eriostachya* (Benth.) Benth. (LABIATAE), Voucher 76. ‘Thupme’ (Gurung), ‘Chirukgherna’ (Amchi).

a) About half spoonful of ground powder (made from leaves, stem and flowers) is taken with a cup of hot water 2-3 times a day for stomachache until recovery.

b) The paste (made from leaves, stem and flowers) is applied on wounds, boils, and other skin diseases two times a day until recovery.

c) About one-forth of a spoonful of powder (made from dried leaves, stem and flowers) is applied on an infected tooth once a day before going to bed at night until recovery.

41) *Ephedra gerardiana* Wall. ex Stapf (EPHEDRACEAE), Voucher 110. ‘Somalatha’ (Gurung).

a) The root is ground to make powder. One spoonful of powder is drunk with a cup of boiled water 2-3 times a day after meal for respiratory disease. i.e., asthma, bronchitis to reduce sound production (wheezes or stridor) during breathing, cough and cold, diuretic, dysuria, to stop sweating, and high altitude sickness problems until recovery.

b) The root paste is applied in cuts and wounds two times a day until recovery.

c) About 20g of pounded root is mixed with two cups of water and boiled. Half cup of decoction is drunk two times a day for body pain, bone pain, cough and cold, diuretic, dysuria, to stop sweating, high altitude sickness problems and asthma. For the treatment of these diseases it is often mixed with other different kinds of medicine such as *Cordyceps sinensis*, and/or honey in equal amounts.

42) **Euphorbia longifolia* D. Don (EUPHORBIACEAE), Voucher 2018. ‘Dhurbi’ (Gurung), ‘Si’ (Amchi).

a) One-forth of a spoonful of root powder is taken with a cup of boiled water one time a day after meal at night for cough and cold, fever, and/or sinusitis until recovery.

b) The decoction of root is applied on the infected part of the body for skin diseases one time a day at night until recovery.

43) **Fragaria nubicola* Lindl. ex Lacaita (ROSACEAE), Voucher 202. ‘Shafaltang’ (Gurung), ‘Shagi’ (Amchi).

a) About 20g of whole plant parts is pounded on stone slab and boiled it with two cups of water. Half cup of decoction is drunk two times a day for menstrual disorders such as heavy periods (menorrhagia), cough and cold, veins pain, edema (swelling of the body), and numbness of limbs until recovery.

b) One-forth spoonful of powder (made from whole plant) is taken two times a day after meal for the same diseases mentioned above.

44) **Galium boreale* L. (RUBIACEAE), Voucher 123. ‘Mara’ (Gurung).

a) The paste of whole plant parts is applied around boils and is said to remove pus and quicken the healing process, two times a day until recovery.

45) *Gentiana robusta* King ex Hook. f. (GENTIANACEAE), Voucher 170. ‘Kiyce’ (Gurung), Kicchakarba’ (Amchi).

a) About 10g of leaves and flowers were boiled on two cup of water and half cup of decoction is drunk two times a day after meals for stomachache, fever, and edema (swelling of the body) until recovery.

b) The paste of flowers is applied on cuts and wounds, boils, and edema (swelling of the body) two times a day until recovery.

46) **Geranium donianum* Sweet (GERANIACEAE), Voucher 153. ‘Kagheshurti’ (Gurung).

a) The plant part (leaves and flowers) are collected, rubbed in the palm of the hand by the thumb and put in a smoking ‘hukkha’ (pipe). The smoke of the ‘hukkha’ is taken twice a day for gingivitis, toothache and to reduce the pain in the teeth.

47) **Gynura nepalensis* DC. (COMPOSITAE), Voucher 309. ‘Mendho’ (Gurung).

a) The fresh latex of the plant can be used to stop bleeding of fresh cuts. The latex cannot be stored, and is used fresh each time.

48) **Heracleum candicans* Wall. ex DC. (UMBELLIFERAE), Voucher 527. ‘Tokar’ (Gurung).

a) About 10g of root is boiled on two cups of water and half cup of decoction is drunk two times a day after meal to reduce blood pressure, bone diseases (‘*Haddhi ko roag*’), stomachache, diarrhoea, dysentery, and joint pain until recovery.

b) Half spoonful of root powder is mixed with a cup of water, and taken 2 times a day after meal for the same diseases mentioned above.

c) The paste of root is applied for wounds, boils, skin diseases, and blisters, two times a day until recovery.

49) *Hippophae salicifolia* D. Don (ELAEAGNACEAE), Voucher 2246. ‘Tarbu’ (Gurung).

a) Half cup of fruit juice extract is mixed with one cup of water and drunk two times a day for cough and cold, chest pain, stomachache, diarrhoea, dysentery, worms, rheumatism and gastritis. The symptom of gastritis includes vomiting, stomachache, and loss of appetite.

b) About one cup of fruit is boiled on two cups of water and reduced it to a cup and half cup of decoction is drunk two times a day until recovery for the same diseases mentioned above.

50) *Hippophae tibetana* Schleecht. (ELAEAGNACEAE), Voucher 114. ‘Tarbu’ (Gurung).

a) Half cup of fresh juice of fruit is drunk by mixing with one cup of water two times a day for diuretic, tonic, cough and cold, enervate period (weakness periods), and to treat worms until recovery.

b) Half cup of fruit is boiled on two cups of water and reduced it to a cup of water; and half cup of decoction is drunk one time a day after meal for the same diseases mentioned above.

51) *Hyoscyamus niger* L. (SOLANACEAE), Voucher 2236. ‘Lantang’ (Gurung).

a) About one-forth spoonful of powder of flowers is put on fire coal and the smoke is given to the affected part of the teeth for gingivitis and to relief pain in teeth 2-3 times a day until recovery.

b) One-eight spoonful of powder of flowers is put on infected teeth at night until recovery.

c) The dried fresh seeds are mixed with tobacco and smoked for gingivitis and to relief pain in teeth once a day until recovery.

52) **Juglans regia* L. (JUGLANDACEAE), Voucher 2135. ‘Katutun’ (Gurung).

a) One-forth of the ground fruit powder is taken with a cup of hot water one time a day after meal for stomachache by mixing with other medicine (confidential mixture) until recovery.

53) *Juniperus communis* L. (CUPRESSACEAE), Voucher 662. ‘Phar’ (Gurung).

a) The juniper fruits are eaten (approx. 5-10 pieces, fresh or dried) to relieve respiratory complaints, chest pains, lung infection, bronchitis, and other infections of upper respiratory tract once a day until recovery.

b) About 5-10 pieces of dried juniper fruits are ground to make powder and taken with hot water once a day for the same diseases listed above until recovery.

54) **Juniperus indica* Bertol. (CUPRESSACEAE), Voucher 277. ‘For’ (Gurung).

a) Leaves and fruits are ground to make powder. Half spoonful powder is mixed with a cup of milk and drunk two times a day after meal for cough and cold, tonsillitis, headache, malarial fever, neck pain and to reduce blood pressure (symptoms of which are described as headache, and vertigo/dizziness ‘*ringhata lagnu*’).

b) The *Amchi* puts half a spoonful of the powder of leaves and fruits on a glowing red fire coal and the scent is smelt by the patient one time a day for same diseases listed above, until recovered.

55) *Juniperus squamata* Buch.-Ham. ex D. Don (CUPRESSACEAE), Voucher 265. ‘Sukri’ (Gurung).

a) The plant parts (leaves and stems) are cut into small pieces and put on ground inside the shed when the animals are affected by different kinds of insects, scabies and wounds.

56) **Maharanga bicolor* (Wall. ex G. Don) A. DC. (BORAGINACEAE), Voucher 255. ‘Maharangi’ (Nepali).

a) The root is pounded on a stone slab and put on clean cloth and squeezed to take out liquid and one spoonful of liquid is mixed with two spoonful of boiled mustard oil. About 1-5 drops of the infusion (liquid) were put on ear 2-3 times a day for ear pain until recovery.

57) **Maharanga emodi* (Wall.) A. DC. (BORAGINACEAE), Voucher 2071. ‘Maharangi’ (Nepali).

a) The root was pounded on stone slab. 2-3 drops of the juice is mixed with one spoonful of mustard oil and 1-5 drops of infusion were put on ear 2-3 times a day for ear pain until recovery.

58) *Malva verticillata* L. (MALVACEAE), Voucher 156. ‘Tangshang’ (Gurung).

a) The flowers of *Malva verticillata* (Tangshang) are ground to make powder and half spoonful powder of *Malva verticillata* is mixed with half spoonful powder of flowers of ‘langtang’ (*Anisodus luridus*). Then, the mixture is mixed with a cup of milk and taken two times a day for cough and cold, tonsillitis, and headache.

59) **Mentha longifolia* (L.) Huds. (LABIATAE), Voucher 275. ‘Patina’ (Nepali).

a) About 10g leaves are boiled in two cups of water. One cup of decoction is drunk in the morning for cough and cold, tonsillitis, and headache until recovery.

b) Leaves used in home-made pickle/relish regularly to increase and purify the blood.

60) **Mirabilis himalaica* (Edgew.) Heimerl (NYCTAGINACEAE), Voucher 313. ‘Nigghibulung’ (Gurung).

a) About 25g of leaves and flowers are crushed on stone slab and paste is applied around the fractured part of the body once a day until recovery.

61) **Morchella conica* Pers. (MORCHELLACEAE), Voucher 331 ‘Guchhichaue’ (Gurung).

a) Eaten whole parts of mushroom as a vegetable for stomachache, wound healing and as a general tonic.

b) 10g of dried ground powder of the dried mushroom is boiled with a cup of water and taken as soup once a day for the same diseases mentioned above.

62.**Morina polyphylla* Wall. ex DC. (DIPSACACEAE), Voucher 2079. ‘Changshar’ (Gurung).

a) Half spoonful of ground root powder is taken with a cup of hot water two times a day after meal for edema (swelling of the body), stomachache, headache, diarrhoea, dysentery, to stop bleeding during child birth, body pain, and numbness of limbs, until recovery.

b) Half spoonful of pounded root is boiled with two cup of water until becoming one cup. One-forth cup of decoction is drunk one time a day at night after meal for the same diseases mentioned above.

63. *Myricaria rosea* W.W. Sm. (TAMARICACEAE), Voucher 320 ‘Angmeo’ (Gurung).

a) About 20 g of pounded mixture of leaves stems and flowers is boiled with 2 cups of water. 2-5 spoonfuls of decoction is drunk with one cup of milk, 2-3 times a day after meal for respiratory disease such as asthma. The symptoms of respiratory diseases include breathing difficulty, bronchitis, and to reduce sound production (wheezes or stridor) during breathing

b) Half spoonful powder of leaves, stems and flowers is mixed with a cup of boiled water, and drunk 2-3 times a day after meal for respiratory diseases mentioned above.

c) Half kg of leaves, stems and flowers are cut into small pieces and cooked it with 10 Litres of water, one kg of wheat flour, and some salt for half an hour. This is given once a day to the animals for respiratory diseases mentioned above.

64.**Nardostachys grandiflora* DC. (VALERIANACEAE), Voucher 256. ‘Panghphoie’ (Gurung).

a) The root of the plant is ground to make powder. Half spoonful of powder is mixed with half spoonfuls of *Aconitum naviculare* ‘Ponkar’, and *Betula utilis* ‘Buspath’ and mixed with three spoonful of ‘chauri ghee’ (butter from a female yak). Then the mixture is taken two times a day with a cup of boiling water for diarrhoea and fever until recovery.

b) One spoonful of root powder is put on red fire coal and the scent is used for conjunctivitis (eye swollen, red and ‘dirty’) at night until recovery.

c) Half spoonful of root powder is taken with a cup of hot water two times a day after meal for gastritis, headache, anthelmintic, edema (swelling of the body), dyspepsia, and rib pain ‘*kokho dukhnu*’.

65. *Neopicrorhiza scrophulariiflora* (Pennell) Hong (SCROPHULARIACEAE), Voucher 431. ‘Kutki’ (Gurung).

a) About 10g root is pounded on a stone slab and boiled in a cup of water. Five spoonfuls of this filtered decoction is mixed with a cup of milk 2-3 times a day for fever (typhoid, malarial, and fever with jaundice), diarrhoea, paralysis, stomachache, dyspepsia (indigestion), snake and scorpion sting, and heart diseases until recovery. The symptoms of heart diseases include continuous pain in the chest and heart, increase heart beat, and difficulty in breathing.

b) The paste of root is applied on cuts and wounds, boils, scabies, ringworm, snake and scorpion stings until recovery.

c) Half spoonful of root powder is mixed with two spoonful of ‘chauri ghee’ and taken 2-3 times a day until recovery for the same diseases listed above.

66.**Onopordum acanthium* L. (COMPOSITAE), Voucher 165. ‘Mangh’ (Gurung).

a) About 15g of root is pounded on stone slab and boiled with two cups of water. Then the half-cup of decoction is drunk two times a day after meal for dysuria and diuretic until recovery.

b) One spoonful root powder is mixed with a cup of boiled water, and drunk two times a day after meal for dysuria and diuretic until recovery.

67.**Origanum vulgare* L. (LABIATAE), Voucher 2224. ‘Akhebobo’ (Gurung).

a) About 50g of whole plant parts is pounded on the stone slab and mixed with two cups of water. Boiled it to reduce to a cup, and half cup of decoction is drunk two times a day for high blood pressure, cough and cold, heart diseases, and fever until recovery.

b) One-forth spoonful of whole plant ground powder is taken with a cup of hot water two times a day for the same diseases mentioned above until recovery.

68. *Paris polyphylla* Sm. (LILIACEAE), Voucher 2009. ‘Satuwa’ (Gurung).

a) About one-forth of the ground powder (made from leaves, flowers and stems) is taken with a cup of hot water one time a day before going to bed to treat worms, until recovery. It is necessary to use a tonic while taking this medicine regularly.

69. *Pinus wallichiana* A.B. Jacks. (PINACEAE), Voucher 276. ‘Thansin’ (Gurung).

a) The bark of the plant was cut into pieces and put on the fractured part of the body continuously for about 2-3 months until recovery.

b) Half spoonful powder of the bark is mixed with a cup of milk, and drunk two times a day after meal for tuberculosis up to 1-2 years regularly. The symptoms of tuberculosis include blood vomiting, vertigo/dizziness (‘*ringhata lagnu*’), and headache.

c) Latex is applied on the abrasion part of the skin in the evening continuously until recovery.

70. *Polygonatum cirrhifolium* (Wall.) Royle (LILIACEAE), Voucher 238. ‘Gomesha’ (Gurung).

a) About 10g of pounded whole plant parts is mixed with two cups of water and boiled until the volume is reduced to one cup. Half cup of decoction is drunk 2-3 times a day, for cough and cold, and fever until recovery.

b) Half spoonful powder of whole plants is mixed with a cup of boiled water and drunk 2-3 times a day after meal for cough and cold, and fever until recovery.

c) Half spoonful of powder of whole plants is taken with a cup of milk or boiled water to increase sexual power one time a day before going to bed until recovery.

71. *Rheum moorcroftianum* Royle (POLYGONACEAE), Voucher 257. ‘Khajo’ (Gurung).

a) Stems are made into a pickle by pounding them on stone slab and mixing with chili, salt, and spices. Eaten twice a day with meals, it helps to increase and purify the blood.

72.**Rhododendron anthopogon* D. Don (ERICACEAE), Voucher 210. ‘Palu’ (Gurung), ‘Sangalin’ (Amchi).

a) The plant part (leaves and flowers) are ground to make powder. Half spoonful of powder is drunk with a cup of milk or hot water two times a day after meal to reduce blood pressure, paralysis, pains in limbs, and waist, inflammation of limbs and fever.

b) Half spoonful (leaves and flowers) powder is put on red fire coal and the scent is smelt by the patient in the evening for paralysis, pains in limbs and waist and inflammation of limbs until recovery.

73. *Rhododendron lepidotum* Wall. ex G. Don (ERICACEAE), Voucher 2122. ‘Bhaiunakpo’ (Gurung).

a) Half spoonful of paste (made from fresh leaves and flowers) is taken with a cup of hot water two times a day for purifying the blood until recovery.

b) The plant part (leaves and flowers) are ground to make powder. Half spoonful of powder is taken with a cup of hot water 1-2 times a day for fever, cough and cold, tonsillitis, by mixing with other different medicinal plants of the Himalaya (confidential mixture) until recovery.

74) *Rosa macrophylla* Lindl. (ROSACEAE), Voucher 343. ‘Seghu’ (Gurung).

a) Half spoonful of fruit powder is taken with a cup of hot water two times a day for fever, diarrhoea, dysentery, and cough and cold until recovery.

b) The paste of the fruit pulp is applied on the infected part for boils two times a day until recovery.

75) *Rosa sericea* Lindl. (ROSACEAE), Voucher 102. ‘Sewa’ (Gurung).

a) Half spoonful of fruit powder is drunk with a cup of hot water three times a day for diarrhoea, dysentery, stomachache, dyspepsia, and bile disorders until recovery.

b) Two fresh or air dried petals are mixed with a cup of hot water and given 2-3 times a day as a substitute of tea to reduce blood pressure. Symptoms of high blood pressure often appear after eating too much salt, feeling angry and vertigo/dizziness (‘*ringhata lagnu*’), feeling headache and urine becomes red in colour and with very bad smell.

c) The fruit pulp is separated, dried in sunlight; ground to make fine powder and the powder is put on infected teeth once a day after meal for gingivitis (toothache) until recovery.

76) *Rubus foliolosus* D. Don (ROSACEAE), Voucher 2019. ‘Mapalan’ (Gurung).

a) About 50g of pounded root is boiled on two cups of water and reduced to one cup. Half cup of decoction is drunk for fever, dyspepsia (indigestion), cough and cold, headache, tonsillitis, vertigo/dizziness (‘*ringhata lagnu*’), enervates periods (weakness periods), and those diseases which are not easily cured by other medicine until recovery.

b) One-forth spoonful of root powder is taken with a cup of hot water 2-3 times a day for the same diseases mentioned above until recovery.

77) *Rumex nepalensis* Spreng. (POLYGONACEAE), Voucher 284. ‘Hali’(Gurung), ‘Lungsho’ Amchi).

a) About 10g of pounded root is boiled in a cup of hot water and ten spoonful of decoction is drunk 2-3 times a day after meal for fever.

b) Half spoonful of root powder is mixed with a cup of water, and drunk 2-3 times a day for fever.

c) One -forth spoonful of ground root powder is taken with a cup of hot water two times a day after meal for gingivitis, joint pain, stomachache, cough and cold, until recovery.

78) **Salix serpyllum* Andersson (SALICACEAE), Voucher 2015. ‘Langmanackpo’ (Gurung).

a) Half spoonful of ground powder (made from leaves and stems) is taken with a cup of hot water one time a day after meal for stomachache, diarrhoea, and dysentery by mixing with different kinds of medicinal plants (confidential mixture, no details given) until recovery.

79) *Saussurea auriculata* (DC.) Sch. Bip. (COMPOSITAE), Voucher 283. ‘Ta’ (Gurung).

a) About 10g of leaves and stems are collected and pounded on stone slab. The pounded mixture is used as paste on infected part where blood circulation stops and the paste (on the body) is slightly heated by a source of heat. It helps in blood circulation in cold weather, and is used especially for older people.

80) *Saussurea fastuosa* (Decne.) Sch. Bip. (COMPOSITAE), Voucher 303. ‘Singamindro’ (Gurung).

a) About 10 drops of juice is applied in minor cuts to stop bleeding.

81) *Selinum wallichianum* (DC.) Raizada & Saxena (UMBELLIFERAE), Voucher 435. ‘Bhutkesh’ (Gurung), ‘Tanak’ (Amchi).

a) Half spoonful of powder (made from leaves and flowers) is taken with a cup of hot water two times a day after meal for stomachache until recovery.

b) The paste (made from leaves and flowers) is applied on cuts and wounds two times a day until recovery.

82) *Stellera chamaejasme* L. (THYMELAEACEAE), Voucher 483. ‘Rekemukta’ (Gurung), ‘Rechaya’ (Amchi).

a) About one-forth spoonful of dried root powder with a cup of hot water is used to make a drink to treat infectious diseases (no other detail is given by the healer).

b) The paste of the root is applied to treat pain from swelling due to fractured bone, and used as an antiseptic for open wounds until recovery.

c) Half spoonful of fresh root is boiled with two cups of water and reduced it to a cup. Half cup of decoction is drunk two times a day for fractured bones, and edema (swelling of the body) until recovery.

83. *Swertia ciliata* (D. Don ex G. Don) B.L. Burtt (GENTIANACEAE) Voucher 311. ‘Tiktha’ (Gurung).

a) The whole plant parts are ground to make powder. Half spoonful of powder is mixed with a cup of milk or hot water, 2-3 times a day for fever, jaundice, malarial fever, diabetes (‘*cheene roag*’), cough and cold, and headache until recovery.

b) About 5g of pounded whole plant is mixed with two cups of water and half cup of decoction is drunk two times a day for fever, jaundice, malarial fever, diabetes (‘*cheene roag*’), wounds, cough and cold, and headache until recovery.

84) **Swertia racemosa* (Griseb.) C.B. Clarke (GENTIANACEAE) Voucher 333. ‘Lakhetiktha’ (Gurung).

a) The whole plant is ground it to make powder. Half spoonful of powder is mixed with a cup of milk or hot water, and drunk 2-3 times a day for fever, jaundice, malarial fever, diabetes, cough and cold, and headache until recovery.

b) About 5g of whole plant is pounded and mixed with two cups of water and half cup of decoction is drunk two times a day for fever, jaundice, malarial fever, diabetes (‘*cheene roag*’), wounds, cough and cold, and headache until recovery.

85) **Taraxacum tibetanum* Hand.-Mazz. (COMPOSITAE), Voucher 304. ‘Khurmang’ (Gurung).

a) About half spoonful of powder (made from leaves, stems and flowers) is taken with a cup of hot water two times a day for jaundice fever, fever which comes from inner bone mostly (‘*haddhi bhitra bata joroho aauene*‘), gastritis , vertigo/dizziness (‘*ringhata lagnu*’), until recovery.

86) **Taxus wallichiana* Zucc. (TAXACEAE), Voucher 2184. ‘Silingi’ (Gurung).

a) The plant powder (made from leaves and stems) is taken with a cup of hot water, two times a day after meal for cancer until recovery. The healers (*amchis*) treatment is confidential (no detailed given by the healers).

87) **Thalictrum cultratum* Wall. (RANUNCULACEAE), Voucher 121. ‘Nagghunensa’ (Gurung), ‘Aotin chauque’ (Amchi).

a) About 10g of leaves and flowers is boiled on two cups of water and half cup of decoction is drunk two times a day after meal for fever.

b) About half kg of leaves and flowers are mixed with one kg of wheat flour and for 20 Litres of water, cooked for half an hour and given to animals (horse, yak, and mule) for diarrhoea.

c) The paste of leaves and flowers is applied for boils, wounds and other skin diseases (ringworm, and blister) two times a day until recovery.

88) *Thymus linearis* Benth. (LABIATAE), Voucher 126. ‘Akhino’ (Gurung), ‘Macto’ (Amchi).

a) About 10g of leaves, stems and flowers of plant is boiled in two cups of water and half cup of decoction is drunk at night for eye infection i.e., eye pain, and conjunctivitis (eye swollen, red and ‘dirty’). It helps older people to see well.

b) The dried plant parts i.e., leaves, stems and flowers is ground to make powder and half spoonful of powder is drunk with a cup of boiled water two times a day after meal for eye infection i.e., eye pain, and conjunctivitis (eye swollen, red and ‘dirty’). It helps older people to see well.

c) Half spoonful of powder from leaves stems and flowers is taken with a cup of hot water two times a day after meal for gingivitis, to increase blood (during weakness periods), and dyspepsia until recovery.

89) **Valeriana jatamansii* Jones (VALERIANACEAE), Voucher 2072. ‘Nappu’ (Gurung).

a) Half spoonful of root powder is taken with a cup of boiled water two times a day after meal for eye pain, and conjunctivitis (eye swollen, red and ‘dirty’), headache, infected wounds, stomachache, cough and cold, and tonsillitis, until recovery.

90) *Verbascum thapsus* L. (SCROPHULARIACEAE), Voucher 195. ‘Yugisingh’ (Gurung).

a) Half spoonful of flower and leaves powder is taken two times a day after meal with a cup of hot water for cuts and wounds, urinary diseases (diuretic, and dysuria) and edema (swelling of the body) until recovery.

91.**Zanthoxylum armatum* DC. (RUBIACEAE), Voucher 2183. ‘Prumo’ (Gurung).

a) The fruits are made into a pickle and eaten to treat cough and cold, tonsillitis, headache, fever, high altitude sickness, numbness of limbs, vertigo/dizziness (‘*ringhata lagnu*’), diarrhoea, and dysentery two times a day until recovery.

b) Dried fruits (excluding seeds) are powdered and one-forth spoonful of powder is taken with a cup of boiled water for diarrhoea, dysentery, and stomachache until recovery.

c) The fruits (excluding seeds) are chewed and put between teeth for gingivitis and to relieve tooth pain.
